# Widely used, short 16S rRNA mitochondrial gene fragments yield poor and erratic results in phylogenetic estimation and species delimitation of amphibians

**DOI:** 10.1186/s12862-022-01994-y

**Published:** 2022-03-28

**Authors:** Kin Onn Chan, Stefan T. Hertwig, Dario N. Neokleous, Jana M. Flury, Rafe M. Brown

**Affiliations:** 1 Lee Kong Chian Natural History Museum, Faculty of Science, National University of Singapore, 2 Conservatory Drive, Singapore, 117377 Singapore; 2 Naturhistorisches Museum der Burgergemeinde Bern, Bernastrasse 15, 3005 Bern, Switzerland; 3 Institute of Ecology and Evolution, University of Bern, Baltzerstrasse 6, 3012 Bern, Switzerland; 4 Leibniz-Institute for the Analysis of Biodiversity Change, Zoological Research Museum Alexander Koenig, Adenauerallee 160, 53113 Bonn, Germany; 5 Department of Ecology and Evolutionary Biology, Biodiversity Institute, University of Kansas, 1345 Jayhawk Blvd, Dyche Hall, Lawrence, KS 66045 USA

**Keywords:** Branch support, Genetic distance, p-distance, Missing data, Phylogenetics, Species delimitation, Systematics

## Abstract

**Background:**

The 16S mitochondrial rRNA gene is the most widely sequenced molecular marker in amphibian systematic studies, making it comparable to the universal *CO1* barcode that is more commonly used in other animal groups. However, studies employ different primer combinations that target different lengths/regions of the 16S gene ranging from complete gene sequences (~ 1500 bp) to short fragments (~ 500 bp), the latter of which is the most ubiquitously used. Sequences of different lengths are often concatenated, compared, and/or jointly analyzed to infer phylogenetic relationships, estimate genetic divergence (*p*-distances), and justify the recognition of new species (species delimitation), making the 16S gene region, by far, the most influential molecular marker in amphibian systematics. Despite their ubiquitous and multifarious use, no studies have ever been conducted to evaluate the congruence and performance among the different fragment lengths.

**Results:**

Using empirical data derived from both Sanger-based and genomic approaches, we show that full-length 16S sequences recover the most accurate phylogenetic relationships, highest branch support, lowest variation in genetic distances (pairwise *p*-distances), and best-scoring species delimitation partitions. In contrast, widely used short fragments produce inaccurate phylogenetic reconstructions, lower and more variable branch support, erratic genetic distances, and low-scoring species delimitation partitions, the numbers of which are vastly overestimated. The relatively poor performance of short 16S fragments is likely due to insufficient phylogenetic information content.

**Conclusions:**

Taken together, our results demonstrate that short 16S fragments are unable to match the efficacy achieved by full-length sequences in terms of topological accuracy, heuristic branch support, genetic divergences, and species delimitation partitions, and thus, phylogenetic and taxonomic inferences that are predicated on short 16S fragments should be interpreted with caution. However, short 16S fragments can still be useful for species identification, rapid assessments, or definitively coupling complex life stages in natural history studies and faunal inventories. While the full 16S sequence performs best, it requires the use of several primer pairs that increases cost, time, and effort. As a compromise, our results demonstrate that practitioners should utilize medium-length primers in favor of the short-fragment primers because they have the potential to markedly improve phylogenetic inference and species delimitation without additional cost.

**Supplementary Information:**

The online version contains supplementary material available at 10.1186/s12862-022-01994-y.

## Background

Over the last four decades, mitochondrial DNA (mtDNA) has been the most commonly used molecular marker in the field of animal systematics and has played a major role in revolutionizing molecular systematics [1–6]. Even in the age of genomics, mtDNA-driven systematic research remains relevant, especially for generating preliminary, large-scale phylogenies and/or species discoveries [7–11] for which the cost of collecting homologous genome-scale data for high numbers of samples is still prohibitive.

To facilitate species identification, accelerate DNA-based taxonomy, and reduce cost, partial fragments of single-locus mtDNA markers were developed to serve as DNA barcodes for animals at the species level. These relatively short fragments were initially promoted as practical resources to aid taxon identification [12] but their utility has since been extended to molecular evolution [13], delineation of species boundaries [14–20], and inference of evolutionary relationships [21, 22]. In eukaryotes, a ~ 650 bp fragment of the mitochondrial cytochrome *c* oxidase subunit 1 (*CO1*) is widely considered to be the universal barcoding gene [12]. Although efforts have been made to utilize the *CO1* barcode in amphibians [23–25], the mitochondrial 16S ribosomal RNA (rRNA) gene has been demonstrated to perform better [22, 26, 27] and is thus, more widely sequenced compared to *CO1.* The 16S rRNA gene is also preferred in other taxonomic groups such as gastropods [28] and hydrozoans [29].

With a total length of approximately 1500 bp, the 16S rRNA gene is the most extensively sequenced gene region in amphibians. However, relatively few studies sequence the entire gene region, and different partial fragments that vary in length and region have been utilized. Sequencing the entire 16S gene region requires the use of multiple primer combinations and usually includes the flanking tRNAs and adjacent 12S gene [30, 31]. Other studies sequence a medium-length ~ 800 bp fragment (e.g., [32, 33]), while the majority of studies sequence a short ~ 500 bp fragment (e.g., [19, 26, 34–41]). Sequences of differing lengths are widely used (often concurrently) to infer phylogenies, delimit species, and serve as proxies of genetic divergence (calculation of *p*-distances) to justify the recognition of new species, making it by far, the most influential gene region in amphibian systematics [8, 11, 19, 33]. However, despite their ubiquitous use, the consistency and relative performance of the different 16S fragments has never been empirically evaluated in vertebrates. As such, it remains untested whether the inconsistent use of different 16S fragment lengths can produce erratic results such as conflicting phylogenetic topologies, variable heuristic branch support, unreliable estimates of genetic divergence among clades, and/or inaccurate estimates of species diversity; all of which may have cascading ramifications for taxonomy, evolutionary inferences, and conservation.

Here, we compare the relative performance of the most widely used 16S rRNA mitochondrial gene regions—short (~ 550 bp), medium (~ 800 bp), and full (~ 1500 bp) sequence lengths. Using the genomic study by [33] as a benchmark, we test topological accuracy, overall quality of branch support, genetic divergence estimates, and quality of species delimitation partitions to determine whether (i) different sequence lengths produce congruent results and, if not, (ii) which fragment yields more accurate results.

## Results

**Fig. 1:**
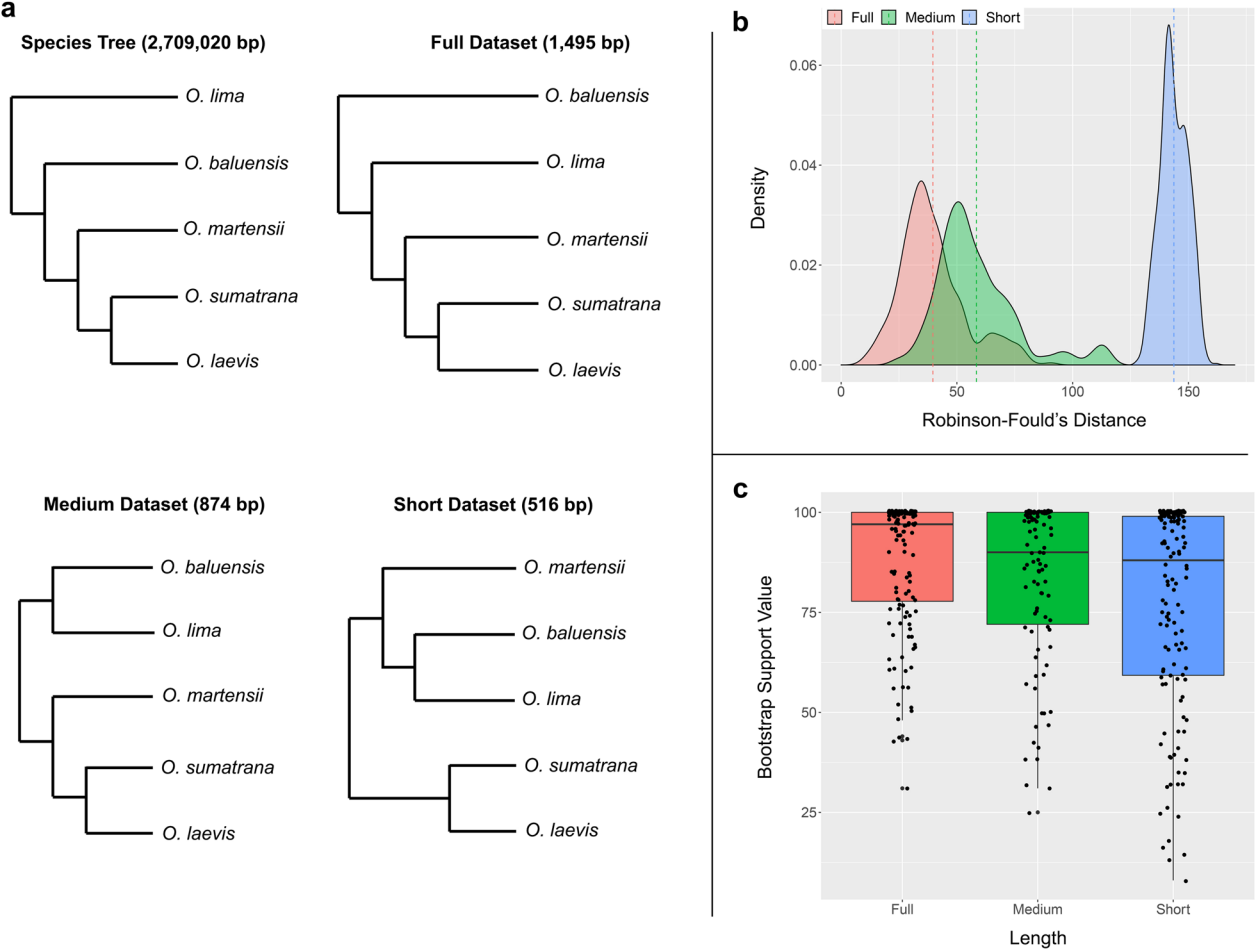
Dendrograms depicting the phylogenetic relationships of nominal *Occidozyga* species. The species tree is based on the genomic study by [33] (**a**). Kernel density distributions of Robinson–Fould’s distances between bootstrap replicate trees and the maximum likelihood tree for each dataset (**b**). Boxplots of bootstrap support values from consensus maximum likelihood trees of the Full, Medium, and Short datasets (**c**)

Summary statistics for each dataset are presented in Table 1. The Full dataset contained the most number and highest proportion of parsimony-informative sites (no. PIS = 736; prop. PIS = 0.49) compared to the Medium (no. PIS = 416; prop. PIS = 0.47) and Short datasets (no. PIS = 239; prop. PIS = 0.46). All three datasets produced markedly different topologies and the only common relationship among all datasets was the sister relationship between *Occidozyga sumatrana* and *O. laevis* (Fig. 1a). Topologies from the Full and Medium datasets were the most similar to the species tree from [33] with only minor differences in the placement of *O. baluensis* and *O. lima*, whereas the topology from the Short dataset was the most dissimilar to the species tree (Fig. 1a). Topological differences were objectively compared against the species tree using RF distances, where the Full and Medium datasets had an RF distance of 2.0, while the Short dataset had an RF of 4.0. The level of incongruence between bootstrap replicates and the inferred maximum likelihood tree was the lowest in the Full dataset (mean RF = 39.6), followed by the Medium dataset (mean RF = 58.4), and the Short dataset (mean RF = 143.7) (Fig. 1b). This pattern of incongruence was reflected in the bootstrap support values of the consensus trees that were highest in the Full dataset (mean = 87.8; median = 97; standard deviation = 16.6) followed by the Medium dataset (mean = 83.1; median = 90; standard deviation = 20.4), and the Short dataset (mean = 76.9; median = 88; standard deviation = 25.8) (Fig. 1c). See Additional file 1 for complete phylogenies with branch support.

**Table 1:** Summary statistics for each dataset (Full, Medium, Short) and pairwise comparisons of uncorrected *p*-distances illustrated in Fig. 3

	Full	Medium	Short
Summary			
Length	1495 bp	874 bp	516 bp
No. variable sites	835	476	266
No. PIS	736	416	239
Proportion PIS	0.49	0.47	0.46
Pairwise *p*-distances			
* Baluensis* vs. cf. *baluensis*	0.093 ± 0.003 (0.088‒0.100)	0.097 ± 0.002 (0.092‒0.101)	0.104 ± 0.003 (0.098‒0.111)
* Rhacoda* vs. cf. *rhacoda*	0.121 ± 0.002 (0.115‒0.124)	0.130 ± 0.003 (0.123‒0.134)	0.124 ± 0.007 (0.116‒0.141)
* Lima* vs. cf. *baluensis*	0.190 ± 0.003 (0.181‒0.200)	0.191 ± 0.004 (0.182‒0.199)	0.186 ± 0.003 (0.177‒0.194)
* Lima* vs. *rhacoda*	0.172 ± 0.006 (0.165‒0.185)	0.169 ± 0.007 (0.162‒0.182)	0.165 ± 0.007 (0.159‒0.182)
* Lima* vs. cf. *rhacoda*	0.166 ± 0.005 (0.155‒0.177)	0.165 ± 0.009 (0.147‒0.182)	0.171 ± 0.012 (0.162‒0.194)
* Martensii* vs. *sumatrana*	0.155 ± 0.005 (0.144‒0.166)	0.154 ± 0.005 (0.146‒0.172)	0.159 ± 0.012 (0.144‒0.194)
* Martensii* vs. *laevis*	0.157 ± 0.009 (0.138‒0.176)	0.157 ± 0.011 (0.130‒0.181)	0.172 ± 0.017 (0.141‒0.202)
* Sumatrana* vs. *laevis*	0.14 ± 0.005 (0.127‒0.160)	0.153 ± 0.007 (0.129‒0.179)	0.135 ± 0.01 (0.101‒0.164)

Values for *p*-distances are average ± standard deviation, followed by Min–Max in parenthesis

*PIS* parsimony-informative sites

**Fig. 2:**
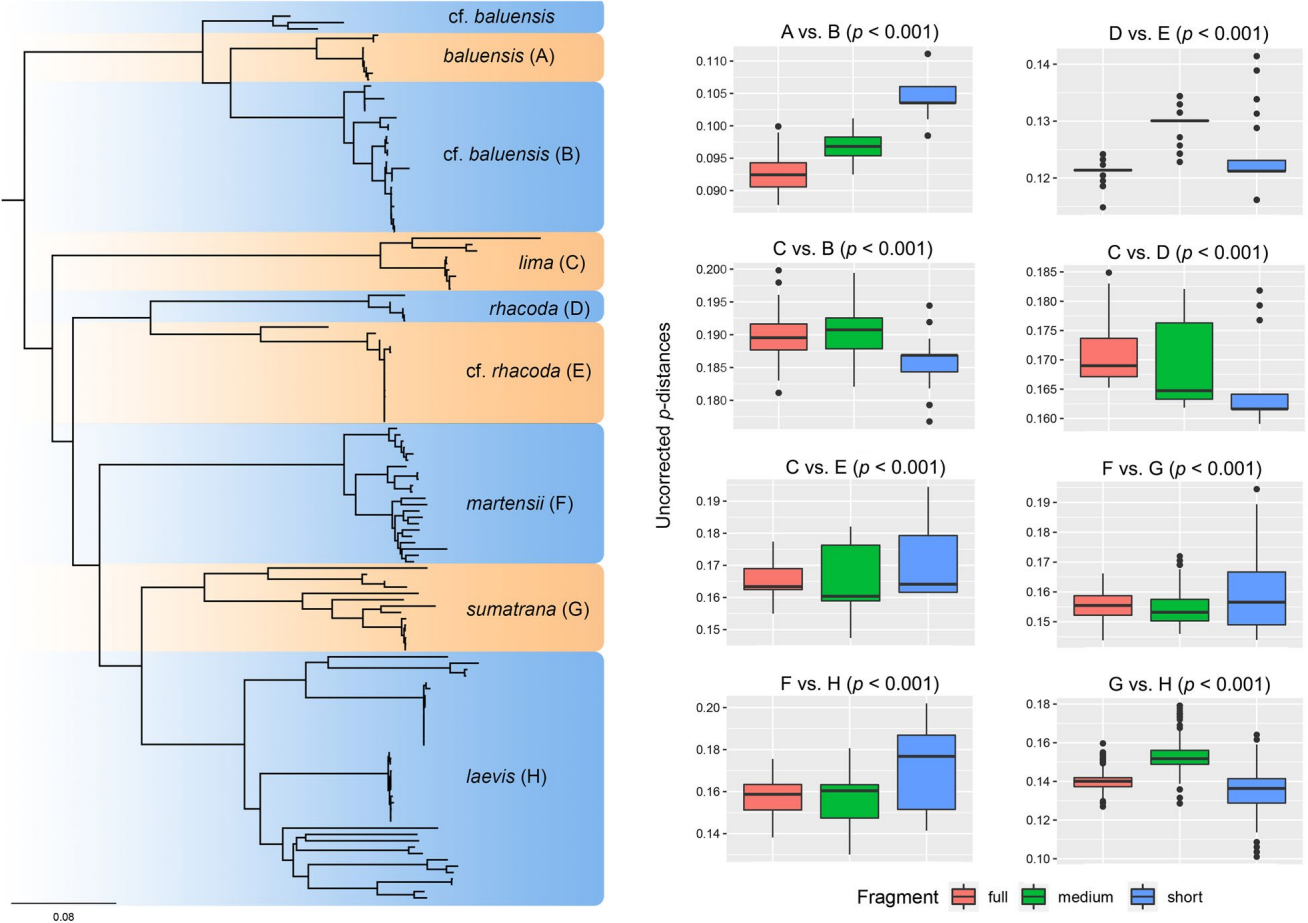
Left: Maximum likelihood consensus tree inferred from the Full dataset. Highlighted clades represent described (**A**, **C**, **D**, **F**, **G**, **H**) and undescribed (**B**, **E**) lineages. Right: Boxplots showing ANOVA *p*-values and pairwise comparisons of uncorrected *p*-distances between closely related lineages

The ANOVA showed that the distribution of *p*-distances among the Full, Medium, and Short datasets were significantly different (*p* < 0.05) across all comparisons (Fig. 2). The magnitude of difference varied among comparisons and ranged up to 3.7% between the Full and Short datasets (Table 1). Although no consistent trend was observed in differences among average *p*-distances, the standard deviation of *p*-distances in the Short dataset was at least as high or more than twice as high as the standard deviations in the Full and Medium datasets. Average *p*-distances were also erratic—the Short dataset produced either higher or lower average *p*-distances compared to the Medium and Full datasets (Fig. 2). The ASAP species delimitation analysis demonstrated that different fragment lengths can yield distinctly different numbers of species partitions (Table 2). The Full dataset produced the lowest optimal number of species (31 species) and the highest distance threshold (0.077) compared to the Medium and Short datasets (37 species; threshold distance = 0.03). More importantly, the ASAP score of the Short dataset was substantially poorer compared to the Long and Medium datasets (Table 2).

**Table 2:** Results of the ASAP species delimitation analysis

Number of species	ASAP score	*p*-val (rank)	W (rank)	Threshold dist
Full				
31	2	2.00e−05 (2)	4.56e−04 (2)	0.077418
36	4.5	7.56e−03 (4)	3.24e−04 (5)	0.04772
59	8.5	1.42e−01 (7)	2.44e−04 (10)	0.014968
32	11.5	1.04e−02 (5)	2.12e−04 (18)	0.069101
26	13.5	1.00e−05 (1)	1.84e−04 (26)	0.094478
Medium				
37	2	7.51e−04 (3)	6.35e−04 (1)	0.039249
36	2.5	1.10e−04 (2)	4.14e−04 (3)	0.06046
39	9.5	5.08e−02 (8)	2.24e−04 (11)	0.034695
27	10	1.00e−05 (1)	1.16e−04 (19)	0.086903
39	10	6.60e−03 (7)	1.63e−04 (13)	0.032463
Short				
37	9	8.57e−04 (2)	2.26e−04 (16)	0.033333
51	9.5	3.73e−01 (14)	3.99e−04 (5)	0.013921
29	11	1.87e−03 (3)	2.18e−04 (19)	0.072876
29	13.5	1.06e−01 (9)	2.23e−04 (18)	0.073809
50	14	5.77e−01 (19)	3.38e−04 (9)	0.014268

A lower ASAP score indicates a better species partition. The ASAP score is the average of ranks from the *p*-val and W parameters combined. *p*-val: probability of panmixia; W: relative gap width metric. See Puillandre et al. [42] for more details

## Discussion

Our results show that full-length sequences of the mitochondrial 16S rRNA gene (Full dataset) not only performed the best across all analyses (topological accuracy, heuristic branch support, *p*-distance distributions, and species delimitation partitions) but also produced significantly different and more accurate results compared to short fragments (Short dataset). The topology inferred by the Full dataset was the most similar to the species tree derived from genomic data, while the topology from the Short dataset was considerably different. Overall branch support for the Short dataset was also lowest, *p*-distances were more variable, and the quality of species delimitation partitions was the lowest (based on the ad hoc ASAP score).

Although recent studies have shown that phylogenetic inference and species delimitation based on the 16S gene can yield erroneous results, those studies were based on data that combined short, medium, and full-length 16S sequences in a single data matrix, resulting in a high proportion of missing data [33, 43]. Surprisingly, the phylogeny inferred from the Full dataset was very similar to the species tree obtained from genomic data (with only minor differences in the placement of *O. lima* and *O. baluensis*) but was markedly different from the 16S phylogeny in [33] that was constructed from a combination of short, medium, and full-length sequences. It is also worth noting that the arrangement of *O. lima* and *O. baluensis* was weakly supported in the genomic species tree, whereas the relationships among all other taxa were strongly supported [33]; thus, uncertainty in the relationships of these two species is not surprising. The relatively poor phylogenetic performance of the short 16S fragment can likely be attributed to insufficient phylogenetic content or to an unfavorable signal/PIS to noise ratio due to strongly deviating substitution rates among the different regions of 16S [44, 45]. Although empirical and simulation studies have shown that the inclusion of taxa with large amounts of missing data in concatenated or supermatrices may not have detrimental effects on phylogenetic accuracy [46–49], this conclusion is only supported if the sequences contain sufficient characters. The phylogenetic placement of sequences with insufficient characters may be random, resulting in poor branch support and reduced accuracy [44], which our results clearly demonstrate (Fig. 1). Taken together, these results indicate that aligning short 16S fragments with longer or complete gene sequences may also result in reduced accuracy as exemplified by [33, 50]. Therefore, the poor performance of short 16S fragments evinced in this study is likely due to insufficient characters, not missing data per se. This is disconcerting because a large portion of published amphibian sequences on public repositories such as GenBank consist of short 16S fragments and many amphibian systematic studies rely solely, or in part, on these short sequences (e.g., [19, 26, 34–41]).

While the full 16S sequence performs best, it requires the use of several primer pairs that increases cost, time, and effort. Our results indicate that the medium-length fragment can be a good compromise: sequencing this region requires only one pair of primers (which can be used for both PCR and also cycle sequencing) and, thus, should cost the same as sequencing the short fragment. The primers for the medium-length sequence are 16SC-L (5′-GTRGGCCTAAAAGCAGCCAC-3′) and 16SD-H (5′-CTCCGGTCTGAACTCAGATCACGTAG-3′) and these have been shown to amplify well across different anuran families [30, 51]. If cost is a limiting factor, our results demonstrate that practitioners should utilize medium-length primers in favor of the short-fragment primers because they have the potential to markedly improve phylogenetic inference and species delimitation without additional cost.

In this study, we have shown how the use of short 16S fragments to calculate uncorrected *p*-distances, so often utilized as evidence to argue for the recognition of new species, can produce variable and inconsistent results (Fig. 2; Table 1). This makes distance thresholds incomparable and untenable for use as criteria to delimit species boundaries unless all comparisons are standardized according to sequence length/gene region, which is not currently the standard practice. Furthermore, explicit species delimitation analysis predicated on short 16S fragments can also yield questionable species partitions. Between the first and second-rank partitions of the ASAP analysis, the number of species inferred from the Short dataset differed considerably (37 vs. 51 species, respectively) despite having only a 0.5 increase in ASAP score. This apparent discrepancy can be attributed to the large differences in ranking of the *p*-val and W parameters that are averaged to form the ASAP score. We interpret this as yet another indication of the erratic and inconsistent property of short 16S fragments when applied to species delimitation analyses. However, despite having better ASAP scores, the number of species partitions inferred by the long 16S fragment remain untenably high. We do not consider this to be a shortcoming of the 16S fragment length per se, but rather, a limitation of single-locus mitochondrial DNA for species delimitation, particulary in cryptic species or continuously occurring populations in which gene flow may be prevalent [33, 43].

Although the 16S gene has been demonstrated to be superior to the universal *CO1* barcoding gene for phylogenetic estimation of amphibians [22, 26, 27], its superiority over *CO1* with regard to non-phylogenetic inferences such as species identification/delimitation is less clear. One study showed that *CO1* outperforms 16S in species identification of hynobiid salamanders [52], while another study reported that 16S had no clear advantage over *CO1* in terms of barcoding of West African frogs [53]. Nevertheless, the 16S gene is clearly efficacious for species identification across numerous anuran taxa [26, 54, 55]. Results from this study also prompt an additional question: is the short fragment of 16S less accurate for species identification compared to longer fragments? Although this question is outside the scope of this present study, we hypothesize that species identification may not, or will be less affected by fragment length because the matching of sequences to taxa typically require fewer sites compared to more demanding analysis such as phylogenetic inference or species delimitation.

## Conclusions

Although short 16S fragments can still be useful for species identification, rapid assessments, or definitively coupling complex life stages in natural history studies and faunal inventories (e.g., genetically identifying tadpoles and assigning them to candidate taxa exemplified among a community or guild of adult frogs), their use as the sole or deterministic source of data in systematic and evolutionary studies should be avoided due to their poor, unreliable, and statistically inconsistent performance. While the full 16S sequence performs best, it requires the use of several primer pairs that increases cost, time, and effort. As a compromise, our results demonstrate that practitioners should utilize medium-length primers in favor of the short-fragment primers because they have the potential to markedly improve phylogenetic inference and species delimitation without additional cost.

## Methods

### Data and study design

We used 147 (seven outgroup and 140 ingroup sequences) published 16S sequences of Puddle Frogs from the genus *Occidozyga* (family: Dicroglossidae). This dataset was chosen for several reasons: (1) it contains dense population/geographic and species-level sampling, which provides a comprehensive representation of genetic variation; (2) full fragment lengths of 16S are available for a broad swathe of operational taxonomic units; (3) this group contains high levels of genetic structure and putative cryptic species, which makes it amenable for species delimitation analyses; and (4) there is a published study on this group based on genomic data (> 6000 loci; 2,709,020 bp), which can serve as a benchmark for our results [33]. Sequences were downloaded from GenBank (Additional file 1: Table S1) and aligned in Geneious v5.6 using the MUSCLE algorithm [56].

**Fig. 3:**
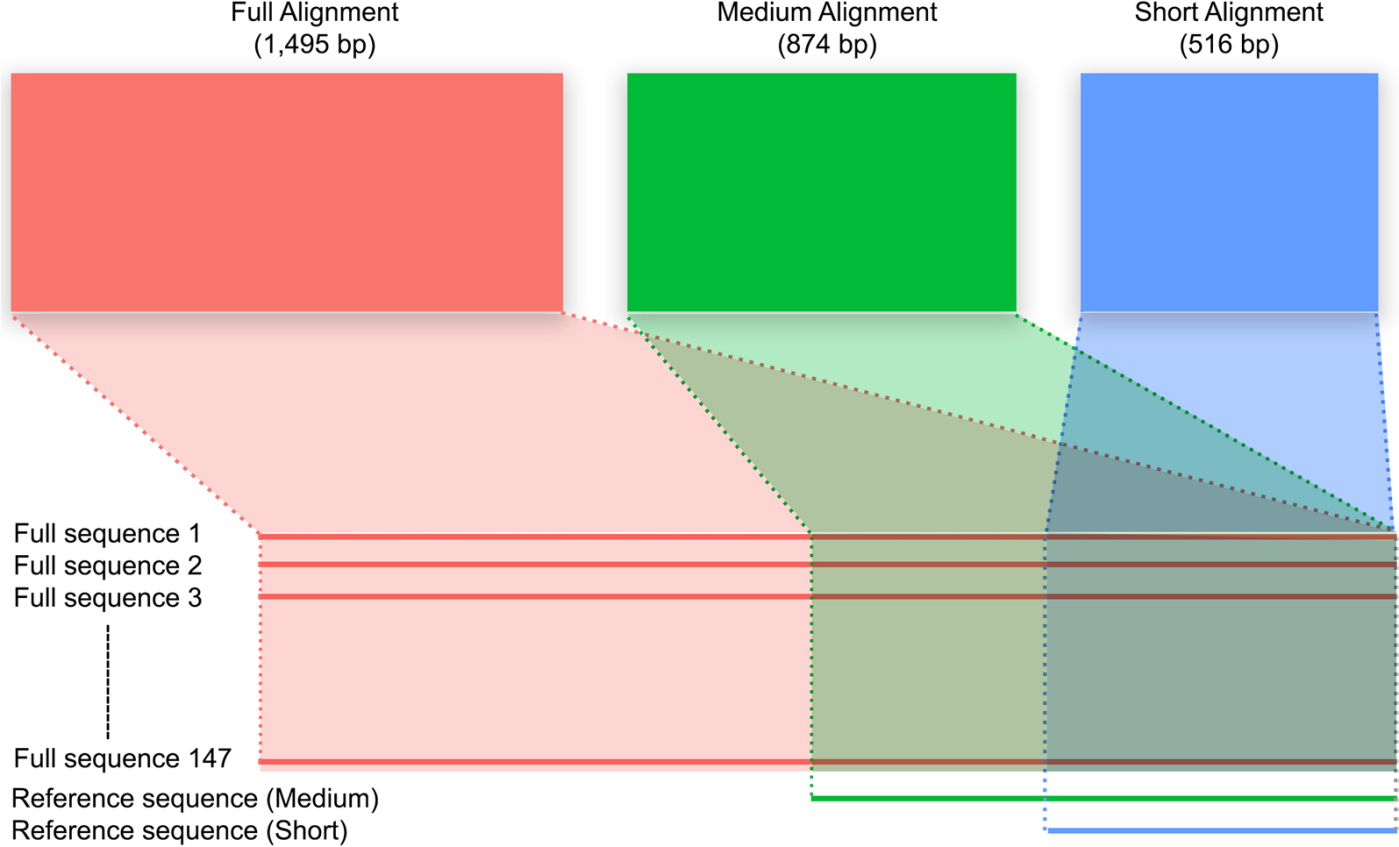
An illustration depicting how the three datasets used in this study were generated. The Full dataset comprised of 147 full-length 16S sequences. The Medium and Short datasets are subsets of the Full alignment and thus, contain the same sequences. Supplementary reference sequences were used to determine the appropriate trimming sites, ensuring that subset alignments were trimmed according to established primer binding sites. Reference sequences were not included in the final datasets as they are not comparable with longer sequences

We generated three separate datasets (i.e., sequence alignments) that contain the same 147 sequences but with each trimmed to different alignment lengths. The first dataset comprised sequences that contain the complete 16S gene region (primers used to sequence the full 16S gene are listed in [57]). After sequence alignment, the adjacent 12S gene and flanking tRNAs were trimmed to ensure that the final alignment only contained the 16S gene region. This trimmed, full-length alignment comprised 1495 bp and is hereafter referred to as the Full dataset. The second and third datasets comprise medium—(Medium dataset; 874 bp) and short-length (Short dataset; 516 bp) sequence alignments. To generate these smaller datasets, we obtained several shorter reference sequences from GenBank and aligned them to the Full alignment to determine the appropriate trimming sites. Medium-length reference sequences were derived from the primers 16SC and 16SD (e.g., [50]), whereas short-length sequences were generated from the primers 16SA-L and 16SB-H (e.g., [26]). These reference sequences were only used to determine the appropriate alignment trimming sites and were not included in the final datasets. This ensures that the Medium and Short datasets contain the same sequences as the Full dataset, but trimmed to shorter lengths based on established and widely used primer combinations (Fig. 3).

### Analysis

In amphibian systematics, the 16S gene is most commonly used to estimate phylogenetic relationships, infer putative species (species delimitation), and justify the recognition of new species via calculations of uncorrected *p*-distances (used as a proxy for genetic divergence). We, therefore, performed these analyses on our Full, Medium, and Short datasets to determine whether different fragment lengths of 16S can result in different estimates of phylogenetic relationships, putative species boundaries, and genetic divergence (uncorrected *p*-distances). For phylogenetic inference, we used IQ-TREE v.1.6 implemented through the IQ-TREE web server [58]. The optimal substitution model was inferred using the “AUTO” function and branch support was assessed using 1000 ultrafast bootstrap replicates [59]. Topological accuracy was determined by comparing the topology of the inferred consensus tree to the species tree obtained from genomic data [33]. The magnitude of topological incongruence was quantified by calculating the Robinson–Fould’s distance (RF) between the consensus mitochondrial trees and the genomic species tree. To facilitate comparisons, clades were collapsed to represent single operational taxonomic units that correspond to nominal species: *Occidozyga baluensis*, *O. lima*, *O. martensii, O. sumatrana,* and *O. laevis* (*O. rhacoda* was not included in the genomic study by [33] and was excluded from this comparison). The quality of branch support was assessed by comparing (i) the distribution of bootstrap values from the consensus trees and (ii) the RF distances between bootstrap replicates and the maximum likelihood tree for each dataset. All RF calculations were performed using the *RF.dist* function implemented in the R package *phangorn* [60].

Uncorrected *p*-distances were calculated in MEGA-X v10.2.3 using the complete deletion option to remove missing data and gaps [61]. Pairwise differences among closely related clades were compared using boxplots and ANOVA to determine whether *p*-distances derived from the Full, Medium, and Short datasets were significantly different. Species delimitation analysis was performed using the program ASAP implemented through the program’s web server (https://bioinfo.mnhn.fr/abi/public/asap/). This program was chosen because it is designed for single-locus data, does not require any a priori knowledge such as the number of species or phylogenetic relationships, performs well under a variety of conditions, and most importantly, produces an adhoc score that can be used to rank and assess objectively the quality of species partitions [42]. The Simple Distance (*p*-distance) model was used to compute distances and all other parameters were left at default values.

## Supplementary Information

**Additional file 1: Table S1.** List of sequences used in this study and their associated GenBank accession numbers.

## Data Availability

All data used in this study are provided either in this document, additional file, or in the supplemental files in the figshare repository (https://doi.org/10.6084/m9.figshare.17292548.v1).
